# Subsurface Bacterioplankton Structure and Diversity in the Strongly-Stratified Water Columns within the Equatorial Eastern Indian Ocean

**DOI:** 10.3390/microorganisms11030592

**Published:** 2023-02-26

**Authors:** Jiaqian Li, Xiuping Liu, Ningdong Xie, Mohan Bai, Lu Liu, Biswarup Sen, Guangyi Wang

**Affiliations:** 1 Center for Marine Environmental Ecology, School of Environmental Science and Engineering, Tianjin University, Tianjin 300072, China; 2 Key Laboratory of Systems Bioengineering (Ministry of Education), Tianjin University, Tianjin 300072, China; 3 Center for Biosafety Research and Strategy, Tianjin University, Tianjin 300072, China

**Keywords:** eastern Indian Ocean, temperature, bacteria, stratification, richness, composition, barrier layer

## Abstract

The consequences of climate change may directly or indirectly impact the marine biosphere. Although ocean stratification has been recognized as one of the crucial consequences of ocean warming, its impacts on several critical aspects of marine microbes remain largely unknown in the Indian Ocean. Here, we investigate the effects of water stratification, in both surface and subsurface layers, on hydrogeographic parameters and bacterioplankton diversity within the equatorial eastern Indian Ocean (EIO). Strong stratification in the upper 200 m of equatorial EIO was detected with evidential low primary productivity. The vertical bacterioplankton diversity of the whole water columns displayed noticeable variation, with lower diversity occurring in the surface layer than in the subsurface layers. Horizontal heterogeneity of bacterioplankton communities was also in the well-mixed layer among different stations. SAR11 and Prochlorococcus displayed uncharacteristic low abundance in the surface water. Some amplicon sequence variants (ASVs) were identified as potential biomarkers for their specific depths in strongly-stratified water columns. Thus, barriers resulting from stratification are proposed to function as an ‘ASV filter’ to regulate the vertical bacterioplankton community diversity along the water columns. Overall, our results suggest that the effects of stratification on the structure and diversity of bacterioplankton can extend up to the bathypelagic zone in the strongly-stratified waters of the equatorial EIO. This study provides the first insight into the effect of stratification on the subsurface microbial communities in the equatorial eastern Indian Ocean.

## 1. Introduction

Global warming changes the water column’s physical and chemical features and can directly or indirectly impact the biosphere in marine ecosystems [1]. Over the past decade, tremendous efforts have been made to understand the impacts of global warming on ocean acidification [2]. However, few studies have focused on the effects of stratification on the marine biosphere [2,3,4,5]. The increased ocean surface temperature stratifies seawater into layers with lighter waters near the surface and denser ones at greater depths. These layers act as barriers to the vertical exchanges of heat, carbon, oxygen, and nutrients [6]. First, stratification has been noticed to impact the primary production and phytoplankton communities. A reduction of primary productivity has been reported over the tropical eastern Indian Ocean [7]. Physically, the dispersal barrier created by strong stratification prevents oxygen from autotrophic microbes in the surface layer from entering the deep water and restructuring the microbial composition [8,9,10,11]. The stratification can directly shallow the mixed layer and sink the deep chlorophyll maximum (DCM) layer, potentially changing the lifestyles of microbes in the euphotic zone [12]. A limited nutrient supply to the surface from the deeper waters due to these physical barriers has been reported to cause the shift of phytoplankton to smaller communities [13]. Thus, it is no surprise that the abundance of picophytoplankton (e.g., picocyanobacteria) is closely related to the strength of stratification in the water column [14]. Furthermore, the ecotype of dominant *Prochlorococcus* has been reported to transform to HLII in a highly stratified layer, whereas the abundance of *Synechococcus* tends to increase under strong stratification conditions [14,15,16]. Such changes in phytoplankton community composition can modify the particulate matter fluxes into the deep waters [17].

Second, hydrological conditions in stratified surface water can potentially influence the community structure along the entire water column [18]. Ocean stratification has been reported to generate isolated patches of turbulence resulting from the velocity shear that often occurs in the stratified regions. Thus, it can consequently modify the properties of particulate fractions [19]. Therefore, it not only changes the quantitative and morphological shifts of surface-derived particulate matter fluxes, but can also alter bacterial communities that are ultimately exported to deep layers [18,20]. The connection among different ocean layers is primarily mediated by particulate matter fluxes from the surface layer [18,21], which acts as a vehicle to transport particle-associated bacteria from the surface to the subsurface layer. Thus, water stratification induced by a rise in temperature can affect the bacterial composition in deep water layers by changing the properties of sinking particles [20,22,23]. The amount of particulate matter exported to the deep water negatively correlates with temperature and surface stratification [24,25]. 

Finally, as stratification is typically observed in the upper 200 m of the ocean, our limited information on the effects of stratification on microbial communities has been largely derived from the surface waters of reported regions [14]. Overall, impacts of stratification on primary production and vertical environmental connections of different layers mostly focus on the surface waters, and the ecological impacts of stratification on the bacterioplankton communities in vertical water columns remain largely unknown [3,26,27]. Notably, the effects of stratification on the vertical diversity of the subsurface bacterioplankton communities need to be elucidated.

As one of the least-sampled oceans, the Indian Ocean has warmed faster than the other tropical ocean basins in the last few decades [28]. The region’s warming-induced stratification has also increased significantly [6]. Notably, surface warming significantly reinforces the stratification in the EIO [29]. With sparse information available on the full vertical structure of the ocean, the effects of stratification on bacterioplankton community in the surface layer (upper 200 m) of this region remain poorly understood. In this study, we collected water samples from multiple depths, ranging from surface to bathypelagic ocean (2000 m), in the stations of equatorial EIO with strong stratification. The main objective was to describe the characteristics of environmental parameters and bacterial distribution under intensely-stratified conditions, aiming to explore the potential response of marine microbes to stratification in the pelagic ocean. Hydrological factors of individual depth were measured in this work, delineating the environmental backgrounds of bacterial communities for further investigation. Vertical patterns of community structure and diversity governed by water stratification were addressed explicitly. This study provides a full-scale vertical view of bacterioplankton community structure in intensely-stratified waters and highlights the potential impacts of stratification on the vertical microbial communities from the surface water to the deep ocean.

## 2. Materials and Methods

### 2.1. Sample Collection and Environmental Analysis

**Figure 1:**
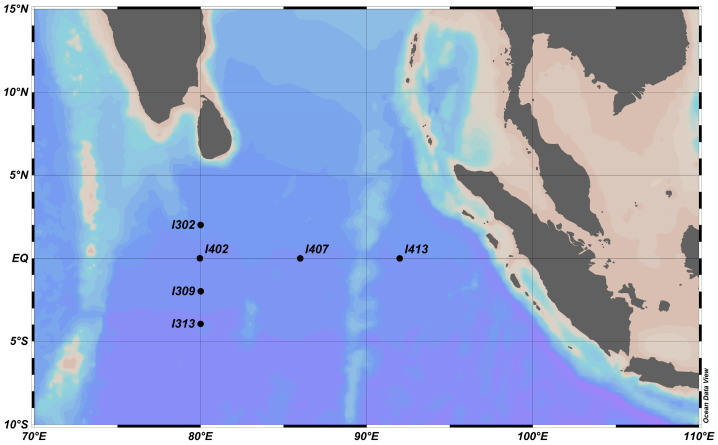
Map of the sampling stations on the equatorial eastern Indian Ocean. Three stations (I302, I309, and I313) were near the equator, and three stations (I407, I407, and I413) were on the equator. Sampling stations were plotted using the Ocean Data View software (https://odv.awi.de, accessed on 3 June 2020).

Sampling was undertaken in the EIO aboard the R/V *Shiyan 3* during an open cruise from 25 March 2018 to 30 April 2018. Six stations were selected to represent the equatorial and surrounding regions of equatorial EIO (Figure 1; Table S1). Water samples were collected from multiple depths ranging from 5 m to 2000 m at each station (Tables S2 and S3). For each depth, 2 L of water sample was filtered through a 0.22-μm polycarbonate filter (Millipore, Billerica, MA, USA) and stored at −80 °C for DNA extraction. A 100 mL of filtrate was transferred to a pre-cleaned polyethylene bottle and stored at −20 °C for nutrient analysis. For bacterial abundance analysis, 4 mL of water sample was mixed with glutaraldehyde (2% *v*/*v*), kept in the dark for 15 to 20 min, and then frozen and stored in liquid nitrogen. Individual samples were diluted (1:10) with a membrane (0.22 μm) filtered TE buffer, stained with 12.5 μL SYBR-I Green solution (1:500 dilution; Molecular Probes^®^), and then incubated in the dark at room temperature for 10 min. Fluorescent beads of 1 μm diameter (Molecular Probes^®^) were added into an individual sample as an internal size standard. The abundance was measured using FACS Calibur Flow Cytometry (BD Biosciences, East Rutherford, NJ, USA) [30].

Details of environmental parameter measurements are provided in our previous study [31]. Briefly, salinity, temperature, dissolved oxygen, and chlorophyll fluorescence were measured with a conductivity-temperature-depth (CTD) system equipped with an oxygen sensor and a fluorometer (Sea-Bird Electronics, Bellevue, WA, USA). Seven inorganic nutrient concentrations were determined using a microplate reader following spectrophotometric methods described in our previous study [31]. Specifically, NH_4_^+,^ NO_2_^−^, NO_3_^−^, SiO_3_^2−^, PO_4_^3−^_,_ total phosphate (TP), and total nitrogen (TN) were monitored in accordance with “Specification for the oceanographic survey” (GB/T 12763.4-2007).

### 2.2. DNA Extraction, Library Preparation, and Sequencing

Genomic DNA was extracted using the E.Z.N.A™ Water DNA Kit (OMEGA Bio-Tek, Norcross, GA, USA) and stored at −20 °C until further use. Primers targeting the V3-V4 hypervariable region 338F (5′-ACTCCTACGGGAGGCAGCA-3′) and 806R (5′-GGACTACHVGGGTWTCTAAT-3′) were used to amplify the target 16S rRNA gene. PCR amplification of the target region was performed in a 10 μL reaction volume with the addition of 5–50 ng template DNA, 0.3 μL of each primer (10 μM), 5 μL KOD FX Neo Buffer (2×), 5 μL of dNTPs (2 mM each), 0.2 μL KOD FX Neo (TOYOBO, Osaka, Japan), and water to the final volume. The PCR program was set to 5 min at 95 °C (initial denaturation), followed by 25 cycles of 30 c at 95 °C, 30 s at 50 °C, and 40 s at 72 °C, and a final extension of 72 °C for 7 min. The purification of PCR products was performed using the VAHTSTM DNA Clean Beads (Vazyme Biotech Co., Ltd., Nanjing, China). The subsequent Solexa PCR reaction was conducted in a 20 μL reaction volume containing 5 μL of purified PCR products, 2.5 μL of each primer (2 μM) with adapters and barcodes, and 10 μL of Q5 HF MM (2×). The PCR program was set to one cycle for 30 s at 98 °C, 10 cycles for 10 s each at 98 °C, 30 s at 65 °C, and 30 s at 72 °C, and final elongation at 72 °C for 5 min. The PCR products were pooled and gel purified using the E.Z.N.A™ Cycle-Pure Kit (OMEGA Bio-Tek, Norcross, GA, USA). High-throughput sequencing of the PCR product was performed on an Illumina HiSeq 2500 platform at Biomarker Technologies (Beijing, China).

### 2.3. Downstream Analysis of Sequencing Reads

The processing and analyses of raw sequencing reads were performed using the QIIME2 microbiome bioinformatics platform [32]. Cutadapt (v1.18) was used to remove the non-biological primer sequences before joining the paired-end sequences [33]. Merged sequences with low quality were subsequently filtered based on the quality score (q score ≤ 20 were removed). Deblur pipeline was used to denoise the data [34] and obtain the ASVs. Samples were rarefied to an equal sampling depth of 11,562 sequences. The ASVs were classified using the silva-138-99-nb-classifier [35]. A total of 5986 ASVs were retained for diversity analysis. 

### 2.4. Determination of Stratification Strength and Mixed Layer Depth 

Gibbs-SeaWater (GSW), a toolbox of MATLAB, was employed to determine the strength of stratification, and the function of SW_BFRQ was picked to calculate the Brunt–Väisälä Frequency Squared (*N*^2^) according to the Equation (1) [6,12]:

(1) $$N^{2}=g\left[-\left(\frac{1}{\rho }\right)\left(\frac{\partial \sigma_{n}}{\partial \sigma_{z}}\right)\right]$$

where *g* refers to the local acceleration of gravity (9.8 m s^−2^), *ρ* denotes the reference seawater density, *σ_z_* is the coordinate of vertical dimension based on the water depth, and *σ_n_* reflects the local potential density depending on the salinity and temperature. The strength of oceanographical stratification was assessed based on the value of *N*^2^. A high value of *N*^2^ indicates stratification in the sampled region with both intensity and stability.

The Isothermal Layer Depth (ILD) and Mixed Depth Layer (MLD), representing the depth of the mixed layer, were calculated from the temperature and density criteria, respectively, following the methods described previously [36].

### 2.5. Statistical Analyses

Alpha diversity indices, namely Shannon diversity, Pielou’s evenness, and observed ASVs, were calculated using the q2-diversity plugin of the QIIME 2 platform. The Kruskal–Wallis test was performed using the alpha-group-significance method of the q2-diversity plugin for pairwise comparisons of alpha diversity indices among different depths. Principal component analysis (PCA) was carried out using the Canoco 5 software to illustrate the linear relationship between environmental factors and sampling depths [37]. Nonmetric multidimensional scaling (NMDS) was employed to visualize the clusters of different sampling depths, which was based on the Bray–Curtis dissimilarities among bacterial communities. Unweighted UniFrac distance generated through the core-metrics-phylogenetic pipeline was applied to evaluate beta-diversity between different test groups, and the significance of differences was tested by PERMANOVA on QIIME 2 platform. The Bray–Curtis dissimilarity matrix was generated using the “vegan” package in R software [38] (https://www.r-project.org, accessed on 11 October 2022). Linear discriminant analysis (LDA) effect size (LEfSe) was performed to determine the differentially abundant ASVs between different depths using the LEfSe tool (http://huttenhower.sph.harvard.edu/galaxy/, accessed on 24 June 2011). The biomarkers at the genus level were identified by analysis of composition of microbiomes (ANCOM) plugin through QIIME 2 platform [39]. 

## 3. Results

### 3.1. Physicochemical Gradients 

**Figure 2:**
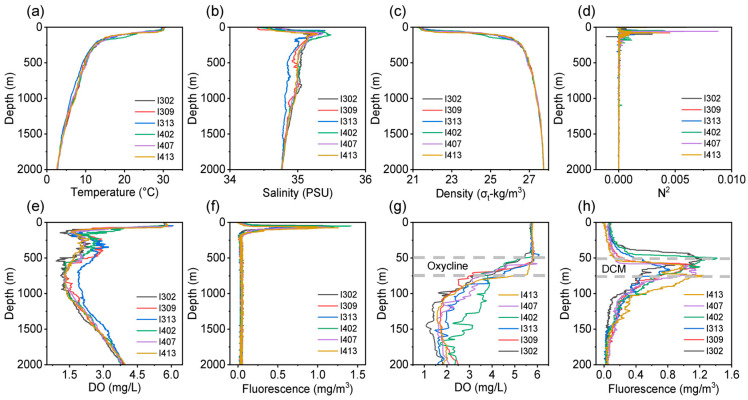
Depth profiles of environmental parameters. (**a**) Temperature (°C), (**b**) Salinity (PSU), (**c**) Density (sigma-t), (**d**) Brunt–Väisälä frequency (N^2^). Depth profiles of Chlorophyll and DO in the (**e**,**f**) entire water column and (**g**,**h**) upper 200 m water column. The region between dashed gray lines represents the deep chlorophyll maximum (DCM) layer and the oxycline layer.

**Figure 3:**
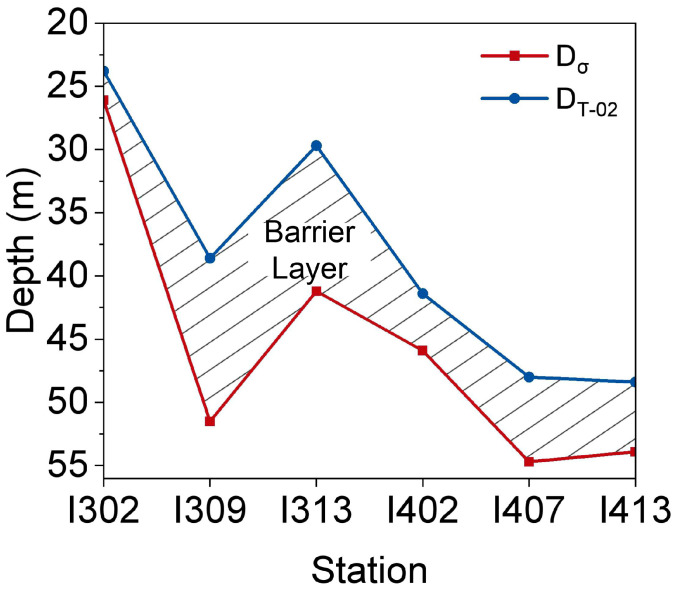
The barrier layer thickness of the equatorial eastern Indian Ocean. The blue line represents the Isothermal Layer Depth (ILD), determined by the definition of the depth where the temperature was 0.5 °C below the surface temperature. The red line represents the Mixed Layer Depth (MLD), defined as the depth surface density plus the density difference brought about by the temperature increment of 0.2 °C. The shaded area represents the barrier layer thickness determined by the difference between the ILD and MLD (D_T-02_-D_s_).

The depth profiles of temperature, salinity, and density indicated a strong stratification in the water column of the equatorial EIO (Figure 2a–c). The sea surface temperature (SST) ranged from 29 °C to 30 °C and salinity from 34.5 PSU to 34.7 PSU. Thermocline, halocline, and pycnocline were detected at depths of 80, 100, and 200 m, respectively (Figure S1a–c). Brunt–Väisälä frequency (N^2^) of the water column was additionally determined to quantify the strength of the stratification (Figure 2d). The maximum values of N^2^ in the upper 200 m of individual stations ranged from 1.7 to 8.8 (×10^−3^) (Figure S1d), suggesting a strong and stable stratification. The ILD and MLD were detected at the average depth of 45.6 m and 38.3 m, respectively (Figure 3). Furthermore, the depth profiles of dissolved oxygen (DO) and chlorophyll indicated the impact of stratification on primary production (Figure 2e,f). The DCM layer ranged from 50 m to 75 m, which overlapped with the depth of oxycline (Figure 2g,h).

**Figure 4:**
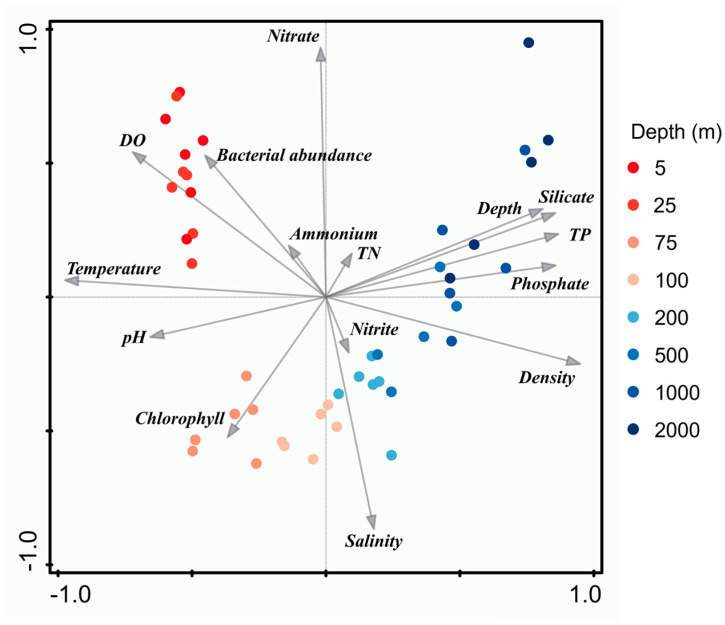
Principal component analysis of measured environmental parameters. Each colored dot represents a sample collected at a specific depth. PC1 and PC2 explained 40.63% and 19.62% of the total variation, respectively.

The PCA analyzed the nutrient concentrations and the abundance of bacterioplankton to further confirm the observation of the stratification. PC1 and PC2 explained nearly 60% of total variations in the environmental data (Figure 4). The PCA indicated that the water column was divided into distinct layers with different ecological characteristics. The temperature was one of the most critical factors driving the sample clustering. The water’s upper 50 m was characterized by higher DO concentration and bacterioplankton abundance, while the water layer from 50 m to 100 m showed higher chlorophyll levels. Water samples collected from 100 m to 1000 m featured high salinity, whereas higher phosphate concentrations, total phosphorus, and distinguished silicate samples from 1000 m to 2000 m. These results further indicated the strong stratification and low nutrient levels in the equatorial EIO stratification.

### 3.2. Vertical Alpha Diversity Patterns of Bacterioplankton 

**Figure 5:**
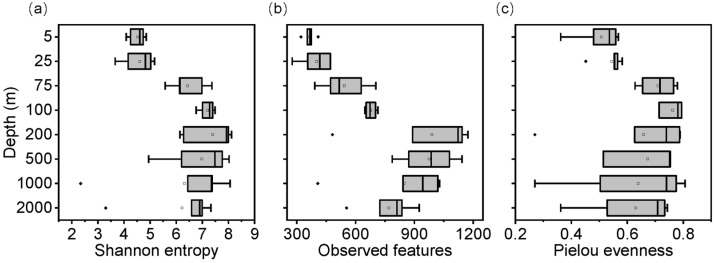
Depth profiles of bacterial alpha diversity indices. (**a**) Shannon entropy, (**b**) Observed features (richness), and (**c**) Pielou’s evenness. The box in the plot represents the interquartile range (IQR), which ranges from the first to third quartiles. The line within the box represents the median. The whiskers extend from the box to the smallest and largest values within 1.5 times the IQR. Any values beyond the whiskers are plotted as individual data points, which may be considered outliers.

The alpha diversity indices, in general, varied significantly along the vertical dimension (*p* < 0.05) (Figure 5). Significant differences in Shannon entropy were observed at the depth above and below 25 m (Figure 5a), likely due to the barrier layer (25–50 m). A similar pattern was also observed with Pielou’s evenness (Figure 5b). In addition, the richness (observed ASVs) varied distinctly with the sampling depth (Figure 5c). Furthermore, the diversity indices among different stations were more variable below 200 m than above 200 m, which indicated horizontal heterogeneity of bacterioplankton communities in the well-mixed layer. Bacterioplankton abundance between stations displayed a greater difference in upper 200 m than that below 200 m, while their diversity inversely exhibited a more pronounced horizontal variation below 200 m (Figure 5 and Figure S2). Moreover, the abundance of bacteria generally decreased with the water depth, whereas the diversity indices were higher below 200 m than above 200 m. Overall, the diversity and abundance of bacterioplankton exhibited opposite trends with depth in both horizontal differences or vertical patterns.

**Figure 6:**
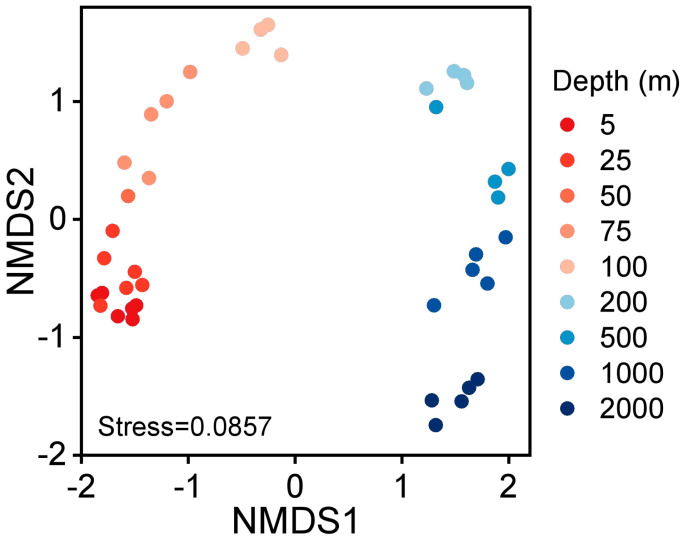
Nonmetric multidimensional scaling (NMDS) plot illustrating the dissimilarities among the bacterial communities of different depths.

The pairwise comparison test results for different diversity indices revealed different patterns (Table S4). The most significant differences in alpha diversity were observed between bacterioplankton communities at the surface waters (5 m and 25 m) and those at the other deeper depths (*p* < 0.05, Kruskal–Wallis test). These findings provide another line of evidence that the barrier layer can be the primary cause of the changes in the diversity of bacterioplankton. Furthermore, the NMDS analysis revealed that bacterioplankton community compositions above and below 200 m depths were quite dissimilar (Figure 6). Particularly, the richness of bacterioplankton below the 200 m was significantly different from that in the upper layer. This observation was consistent with the vertical alpha diversity patterns, with an evidential depth-wise segregation of the bacterioplankton communities under the barrier layer. Thus, these findings support that the barriers resulting from the surface stratification possibly led to the relatively low mixing of bacterioplankton communities in the deeper layers.

### 3.3. Depth-Related Bacterioplankton Community Compositions

**Figure 7:**
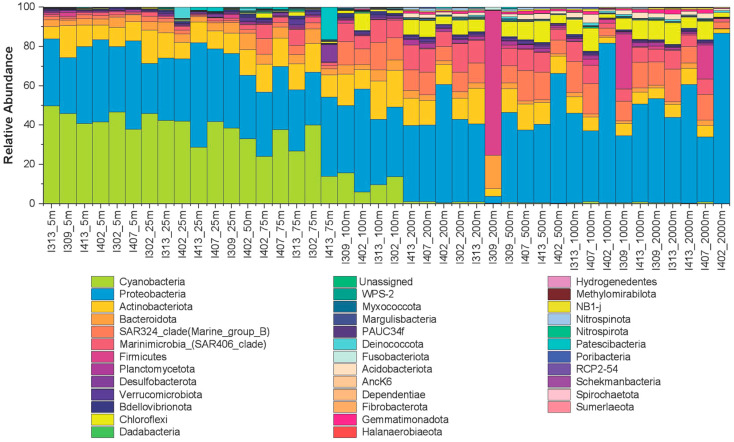
Bar plot showing bacterial community compositions (at phylum level) of different sampling stations.

A total of 36 bacterial phyla were identified in the water column of the equatorial EIO (Figure 7). The phylum Cyanobacteria was predominant at the surface (5–100 m), accounting for 5.96% to 50.13% of the samples. Nearly 33% of total ASVs were assigned to phylum Proteobacteria. This phylum dominated most samples from various depths and stations, accounting for 25.63% to 86.6% of entire sequences. Furthermore, the members of SAR324 and SAR406 (Marinimicrobia) clades were found at deeper depths (below 50 m). The relative abundance of Actinobacteria remained somewhat stable along the water column, with only a slight decrease at 2000 m in all stations. Still, several phyla, such as Desulfobacterota, Firmicutes, and Patescibacteria, were also observed in a few stations. 

The composition of the top 100 ASVs was relatively homogeneous across stations in the surface layer (5–25 m) (Figure S3). However, at the layer of 50–75 m, where primary production was more intense, the composition of these ASVs across stations showed more variations. Significant vertical variations were also observed in the upper and below the 200 m, which suggested that the composition of dominant bacterioplankton can likely be affected by stratification. To further assess the phylogenetic turnover (beta diversity) of community composition between layers with different stratification intensities and a given pair of depths, pairwise comparisons were performed using unweighted Unifrac distance (Figures S4 and S5, Tables S5 and S6). Overall, bacterial composition in the stratified-surface layer (5–200 m) was significantly different from that in the subsurface layer (500–2000 m) (*p* < 0.01, PERMANOVA) (Figure S4, Table S5). Pairwise comparison of bacterioplankton communities revealed significant differences among individual depths (Figure S5, Table S6). Except for the depths 5 and 25 m, the phylogenetic composition of bacterioplankton was significantly different among the other depths.

**Figure 8:**
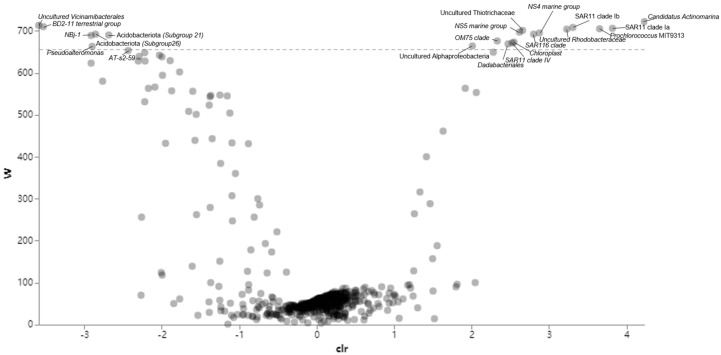
ANCOM generated volcano plots. The W value indicated the number of sub-hypotheses that have passed for a given taxa. Taxa with higher W were more significantly abundant in specific group versus another group. The clr value referred to the effect size change between the compared groups. Genera identified as statistically significant biomarkers were labelled. Positive clr indicated higher abundance in surface layer group against to subsurface layer group.

**Figure 9:**
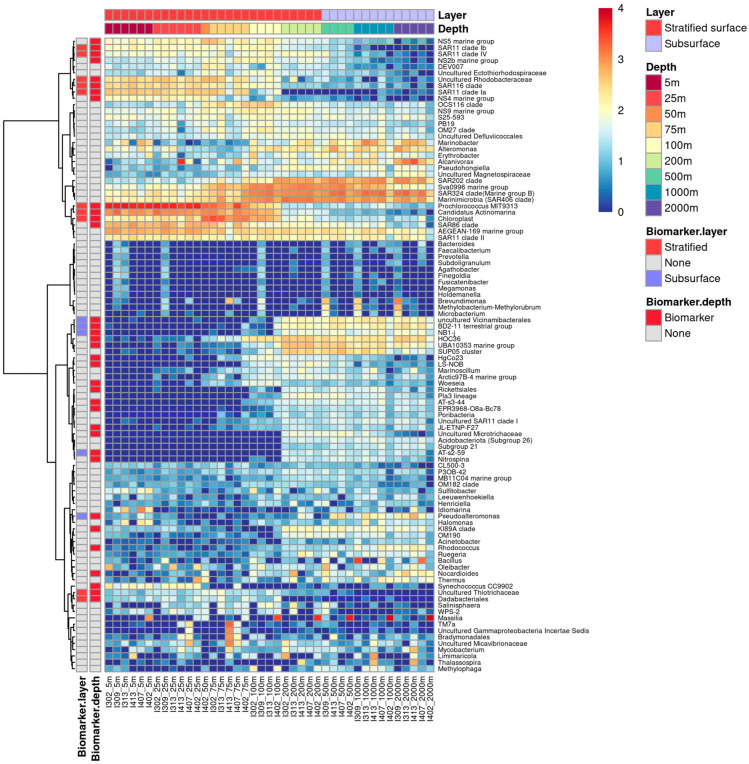
Heatmap of top 100 abundance genus. The relative abundance was log10 transformed and scaled by row. Genera (rows) are clustered by similarity using Ward’s hierarchical agglomerative method and labeled with results of ANCOM. Genera identified as biomarker of depths (*p* < 0.05) were labeled red in the bar of “Biomarker-depth”. Genera identified as biomarker in stratified-surface layer were labeled red, whereas genera identified as biomarker in subsurface layer were labeled blue in the bar of “Biomarker-layer” (*p* < 0.05).

LEfSe revealed that most of the top 100 ASVs dominating the upper 50 m layer were possible biomarkers, suggesting their unique adaptation to the surface layer’s environment. Biomarkers were rarely detected at the 100–200 m layer. Therefore, the barrier layer can be considered as an “ASV filter” that probably acts as a barrier for ASVs to enter the deep layers. To further understand the preference of depths with different stratification intensities, bacterial genera were annotated using ANCOM. Their functions as biomarkers were sorted based on layers (stratified-surface and subsurface layers) (Figure 8) and water depths (Figure 9). A total of 13 genera were detected as a biomarker for the stratified-surface layer (5–200 m), while 8 other genera were specifically abundant within the subsurface layer (500–2000 m) (Figure 8 and Figure 9). Furthermore, a total of 59 genera were identified as a biomarker in individual depths (Table S7, Figure 9).

To better understand the distribution of those genus biomarkers along the water columns, the 100 most abundant bacterial genera are illustrated by the heatmap. The result of ANCOM is labeled (Figure 9). These top 100 genera displayed obvious and interesting vertical distribution patterns. Among all biomarkers identified by ANCOM, a total of 13 genera were simultaneously identified as biomarkers in the stratified-surface layer and other depths (Figure 9), while only 3 biomarker genera occurred in the subsurface layer and depths below it.

## 4. Discussion

### 4.1. Vertical Environmental Patterns and Stratification

This study reveals the vertical variations of several environmental parameters, from surface to bottom (2000 m), in the water columns of equatorial EIO. The environmental patterns observed in this study generally agree with a previous report on the EIO [40]. The depth-related variations of physicochemical parameters observed in this study are similar to the findings of earlier studies on other ocean basins [41,42]. The surface water was characterized by a high temperature and low salinity (Figure S1a,b), indicative of a relatively low-density layer on the pycnocline. Particularly, the temperature was one of the most critical factors driving the sample clustering (Figure 4). In addition, high SST was detected in this study. Although the water temperature of the Indian Ocean is significantly modulated by the interannual coupled ocean–atmosphere phenomena, namely, the Indian Ocean Dipole (IOD) event, the SST in the equatorial EIO is continuously above 27 °C throughout the year [43,44]. Thus, the high SST is generally considered to be the primary factor of stratification in the Indian Ocean [6]. Nevertheless, compared with the temperature and density, the salinity profile presented more distinct variations among stations (Figure S1b), indicating that salinity could be a good indicator of local stratification. However, the stratification reported in other tropical oceans was attributed to the combined effect of temperature, DO, and salinity fluxes [45].

In line with previous studies [12], the Brunt–Väisälä frequency (N^2^) data further provide a theoretical verification of the intensive stratification (Figure 2d and Figure S1d) in the equatorial EIO. The barrier layer with a thickness ranging from 2 m to 13 m also revealed the presence of a stable stratification (Figure 3). Moreover, we found that the thermocline occurred around the depth 70 m, which was shallower than previously reported [44]. The thermocline depth varies seasonally in the Indian Ocean with a strong semi-annual cycle, shifting between a relatively shallow depth (~70 m) and a slightly deeper depth (120 m) [44]. The dramatic temperature change is usually expected to aggravate the stratification, retard vertical mixing, and shallow the mixed layer [46]. Consequently, the vertical transport of nutrients is bound to change and ultimately affect microbial community structure [47,48,49]. In a current study, the DCM layer contained the oxycline (Figure 2g,h), suggesting that a relatively lower primary production characterized the equatorial EIO. This result is coincident with a previous report [50], indicating the stratification in equatorial EIO does inhibit the supplement of nutrients to the euphotic layer. This will potentially cascade through the entire food web in the area [7]. Nevertheless, several other studies conducted in the western Indian Ocean showed increasing trends of net primary production to the enhancing of stratification [51,52], implying that the microbial responses to stratification are complicated and need further research. In this work, the mixed layer was detected at a relatively shallow depth, as reported previously [53]. This finding supports the notion that microbial communities may colonize closer to the surface where radiation is more intense [54].

### 4.2. Impact of Stratification on the Vertical Structure of Bacterioplankton Communities 

This study provides a comprehensive view (surface to 2000 m) of the vertical alpha diversity patterns of bacterioplankton in the water column of the equatorial EIO. The alpha diversity data revealed the lowest diversity, evenness, and richness of bacterioplankton in the water column’s upper surface layer (above 50 m). These findings likely indicate that bacterioplankton diversity might decline in high-temperature conditions. Besides the vertical dimension, a global survey on the dynamics of bacterial richness suggests that species richness increases with the latitude decrease [55], indicating that temperature is one of the most significant factors influencing the richness gradients. Nevertheless, it may be premature to conclude that temperature is the sole factor in diversity reduction; other environmental factors, such as pH, chlorophyll, and DO, can also lead to water stratification. Stratifications due to other factors are equally important in affecting microbial diversity [56,57]. To further understand the complex stratification effects on bacterioplankton diversity, we investigated the alpha diversity patterns from subsurface to deep layers (2000 m). The vertical patterns of alpha diversity indices differed significantly between layers with and without stratification (*p* < 0.05, Wilcoxon signed-rank test/Kruskal–Wallis test) (Figure 5). Similar patterns were detected in the eastern North Atlantic waters [18]. The richness index was more sensitive to depth variations than the Shannon entropy and Pielou’s evenness indices, indicating that richness was likely the main parameter of the bacterioplankton diversity in responding to the stratification. However, the presence of a vertical richness gradient can be alternatively explained by the available energy sources in ambient surroundings, which can determine the fluctuation of species richness [58]. Furthermore, the pairwise test results (Table S4) suggested that bacterioplankton richness in the barrier layer may show an ambiguous relationship with environmental factors. As stratification divides the water column into demarcated layers and generates different environmental conditions, it is reasonable to conjecture that influencing factors of bacterioplankton richness gradients differ within and underneath the stratified water. 

Species evenness has been generally deemed as the indicator of ecosystem functional stability [59] and is usually correlated with water stratification [60]. Bacterioplankton evenness was consistently higher in the water layers from the subsurface to 2000 m than those within the stratified surface layer. Strong stratification has been suggested to decrease the species evenness in surface water through effecting the spatial overlap of phytoplankton, which may directly or indirectly affect primary and/or secondary production [61]. Thus, thermal stratification may indirectly weaken ecological stability. Furthermore, the evenness has been reported to be relatively high in the surface layer of other tropical regions without stratification. The lower evenness in the surface layer of equatorial EIO than in other studies [18,62] provides further evidence for the weakening effects of stratification on species abundance variability.

The vertical composition of bacterial communities shows a visible depth-stratified pattern (Figure 8) which is consistent with the previous studies of other oceanic regions [14,63,64,65,66]. The consistent depth-clustering patterns observed in the NMDS plot (Figure 6) and heatmap (Figure 8) reveal the structuring of the water column into four vertical layers (5–100 m, 200 m, 500–1000 m, and 2000 m). These water layers based on the bacterioplankton community composition somewhat correspond with the traditional classification of ecological regions of the ocean (upper epipelagic, lower epipelagic, mesopelagic, and upper bathypelagic) [67,68]. Our results seem to support that stratification may not shift the selective adaption of bacterioplankton in the ocean’s ecological layers. However, the beta diversity analyses revealed that the significant difference of phylogenetic composition occurred between layers with different stratification intensity and different individual depths.

The homogeneity of bacterioplankton communities throughout the water column significantly depends on their feasibility of passing through the pycnocline. The pycnocline is understood as the main factor that restricts free-living prokaryotes from entering the deep ocean [18]. Interestingly, we observed the presence of ASVs assigned to Prochlorococcus clade MIT9313 in the upper surface layer (5–50 m). Members of this clade were reported to be abundant near the bottom of the euphotic zone with high sensitivity to light fluctuation [69]. This can be explained with the theory that the existence of stable stratification creates multiple spatial niches, with better light competitors at deeper depths co-existing with better nutrient competitors at the surface [70]. Clearly, the results of this study suggest that the effects of surface water stratification on the bacterioplankton communities can extend beyond the surface layer, euphotic zone, and the subsurface layers. Additionally, some bacterioplankton known to diffuse in the surface ocean were detected at low percentages in our study. For example, members of the clades SAR11 and SAR86, which were reported in significant numbers in the oligotrophic oceans [18,71], account for relatively low portions of the entire communities in this study. Interestingly, the low contribution of SAR11 clade in equatorial EIO was previously reported in a metagenomic study conducted in equatorial EIO [40], indicating that the low proportion of SAR11 in this region was not random. Members of these clades are typically enriched in free-living communities [72]. Their relatively low proportions in our study may indicate limited carbon and sulfate cycling in the surface layer. Furthermore, the variations of bacterioplankton community compositions among different layers observed in this study can be explained by the fact that stratification resulted in the reduction of primary production in the equatorial EIO, which may consequently lower the particulate matter fluxes [7]. Interestingly, genera including SAR11 clade Ib, SAR11 Clade IV, SAR116 clade, SAR11 clade Ia, Candidatus Actinomarina, *Dadabacteriales*, *Pseudoalteromonas*, *AT-s2-59,* and uncultured genera of Thiotrichaceae, Vicinamibacterales, Alphaproteobacteria, and Rhodobacteraceae were identified as biomarkers, both in the stratified layer and individual depths. These results implied that these genera were likely sensitive to the stratification gradients in upper water layers. In contrast, only three genera, including Acidobacteriota Subgroup26, *NB1-j*, and Acidobacteriota Subgroup 21, were identified as biomarkers in subsurface water and individual depths. However, most biomarker genera in subsurface water seemed to be more depth-dependent than those in the entire stratified layer. Thus, these biomarker genera likely rely more on resources from individual depth (e.g., particulate matters), rather than light intensity or temperature. Furthermore, results of this study suggest that surface water stratification can potentially attenuate vertical bacterioplankton community distribution of water column and may weaken vertical particulate matter fluxes into the deep ocean.

## 5. Conclusions

This work reports the first investigation of the link between bacterioplankton diversity along the entire water column (surface to 2000 m) and stratification in the equatorial EIO. A strong stratification occurred in the upper 200 m of the water column, and the environmental conditions of deeper layers exhibited a typical depth-related clustering pattern. Bacterioplankton richness in stratified surface and deep subsurface layers was found to be controlled by different environmental factors. Lower bacterioplankton evenness was observed in the surface layer than in other layers, which likely indicated that stratification affected the community stability in the surface layer. Furthermore, our results showed that bacterioplankton community composition was altered to a certain degree in stratified layers, which can impact certain marine ecosystem functions like carbon cycling. Our findings delineate the characterization of equatorial EIO in terms of hydrogeography and bacterioplankton ecology and emphasize the need to clarify microbial responses to stratification. More efforts are still needed to understand the mechanisms of bacterioplankton feedback in deeper water in response to stratification.

## Data Availability

Raw sequencing reads are available in NCBI under the BioProject PRJNA794046. The latest workflows and other ancillary data can be found in the GitHub repository (https://github.com/sujianxin514/euqtorial-EIO-bacteria, accessed on 13 May 2022).

## Supplementary Materials

The following supporting information can be downloaded at: https://www.mdpi.com/article/10.3390/microorganisms11030592/s1, Figure S1: Magnified view of the depth profiles of stratification indicators. (a) Temperature (°C), (b) Salinity (PSU), (c) Density (sigma-t), (d) Brunt–Väisälä Frequency.; Figure S2: Bacterial abundance between different depths. The box in the plot represents the interquartile range (IQR), which ranges from the first to third quartiles. The line within the box represents the median. The whiskers extend from the box to the smallest and largest values within 1.5 times the IQR. Any values beyond the whiskers are plotted as individual data points, which may be considered outliers.; Figure S3: Heatmap of top 100 amplicon sequence variants (ASVs). The relative abundance was log2 transformed and scaled by row. The depths and stations of samples are shown in the top bar. Biomarkers were determined by LEfSe and are shown on the left of the heatmap with the taxonomy at the class level.; Figure S4: Unweighted UniFrac distance between stratified-surface layer (5–200 m) versus subsurface layer (500–2000 m). The distance to (a) stratified surface layer and to (b) subsurface layer were presented as the value of Y-axis. The box in the plot represents the interquartile range (IQR), which ranges from the first to third quartiles. The line within the box represents the median. The whiskers extend from the box to the smallest and largest values within 1.5 times the IQR. Any values beyond the whiskers are plotted as individual data points, which may be considered outliers.; Figure S5. Unweighted UniFrac distance between different depths. The distance of other depths to (a) 5 m, (b) 25 m, (c) 50 m, (d) 75 m, (e) 100 m, (f) 200 m, (g) 500 m, (h) 1000 m, and (i) 2000 m were presented as the value of Y-axis. The box in the plot represents the interquartile range (IQR), which ranges from the first to third quartiles. The line within the box represents the median. The whiskers extend from the box to the smallest and largest values within 1.5 times the IQR. Any values beyond the whiskers are plotted as individual data points, which may be considered outliers.; Table S1: Sampling information about individual stations.; Table S2: Information about sampling depths used in environmental and sequencing analyses.; Table S3: Information about sampling depths.; Table S4: Relationship (Kruskal-Wallis pairwise test) between the bacterial alpha-diversity indices of different depths.; Table S5: Pairwise PERMANOVA results between stratified layer and subsurface layer.; Table S6: Pairwise PERMANOVA results of different depth.; Table S7: Genera identified as biomarker for individual depth.
