# Supplementary material for: Subsurface Bacterioplankton Structure and Diversity in the Strongly-Stratified Water Columns within the Equatorial Eastern Indian Ocean
Source: Microorganisms. 2023 Feb 26;11(3):592. doi: 10.3390/microorganisms11030592 (PMC10058062; doi:10.3390/microorganisms11030592)
Supplement: Supplementary file 1 [file microorganisms-11-00592-s001.zip › microorganisms-2159445-supplementary.pdf]

Supplementary Material

Subsurface Bacterial Structure and Diversity in the Strong Surface Stratification Region within the Equatorial Eastern Indian Ocean

Jiaqian Li [1], Xiuping Liu [1], Ningdong Xie [1], Mohan Bai [1], Lu Liu [1], Biswarup Sen [1,*], and Guangyi Wang [1,2,3,*]

1 Center for Marine Environmental Ecology, School of Environmental Science and Engineering, Tianjin University, Tianjin 300072, China
2 Key Laboratory of Systems Bioengineering (Ministry of Education), Tianjin University, Tianjin 300072, China
3 Center for Biosafety Research and Strategy, Tianjin University, Tianjin 300072, China

* Correspondence: bsen@tju.edu.cn (B.S.); gywang@tju.edu.cn (G.W.)

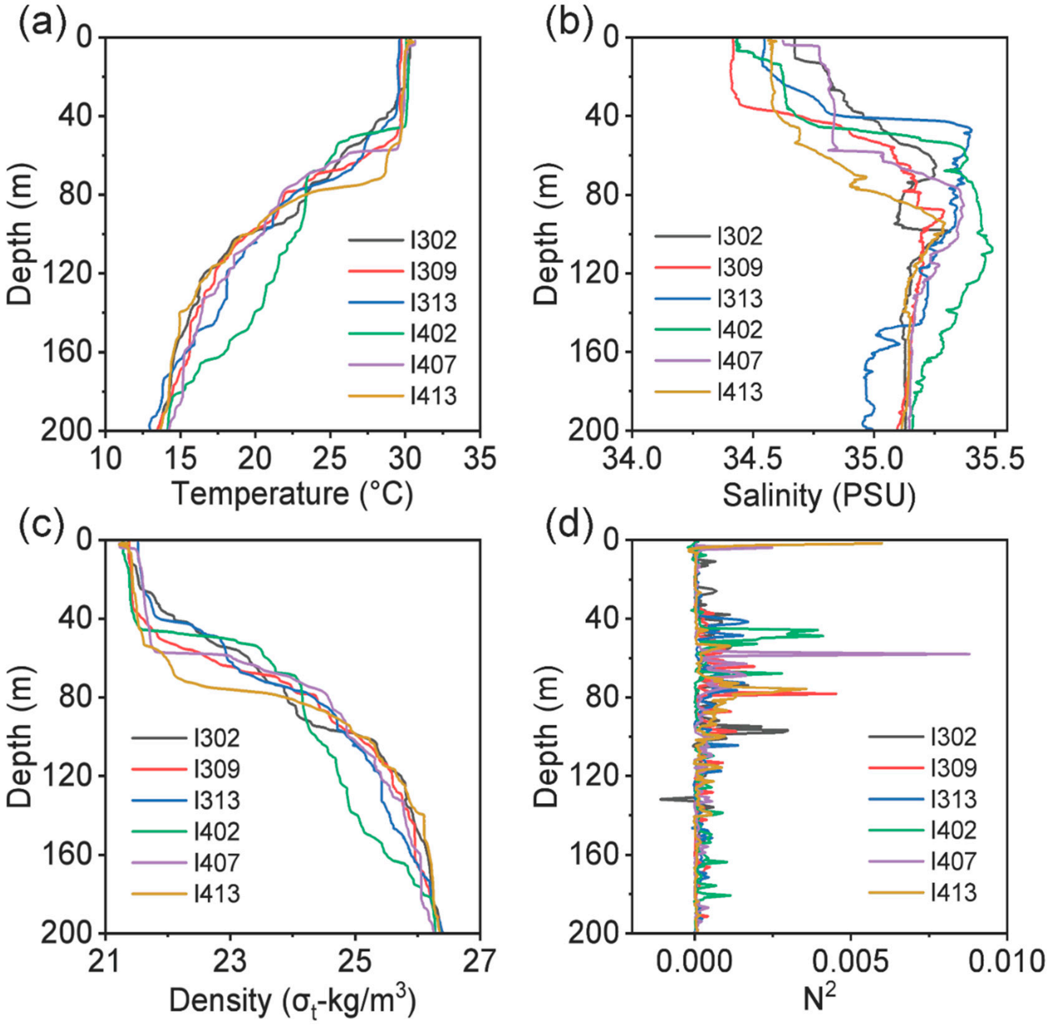

Figure S1. Magnified view of the depth profiles of stratification indicators. (a) Temperature (℃), (b) Salinity (PSU), (c) Density (sigma-t), (d) Brunt–Väisälä Frequency.

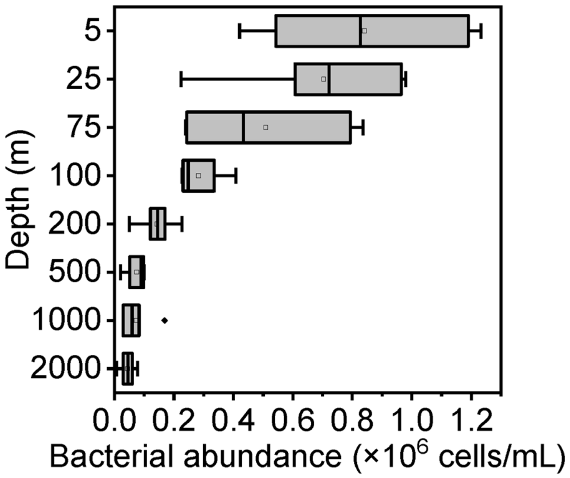

Figure S2. Bacterial abundance between different depths. The box in the plot represents the interquartile range (IQR), which is the range between the first and third quartiles. The box in the plot represents the interquartile range (IQR), which ranges from the first to third quartiles. The line within the box represents the median. The whiskers extend from the box to the smallest and largest values within 1.5 times the IQR. Any values beyond the whiskers are plotted as individual data points, which may be considered outliers.

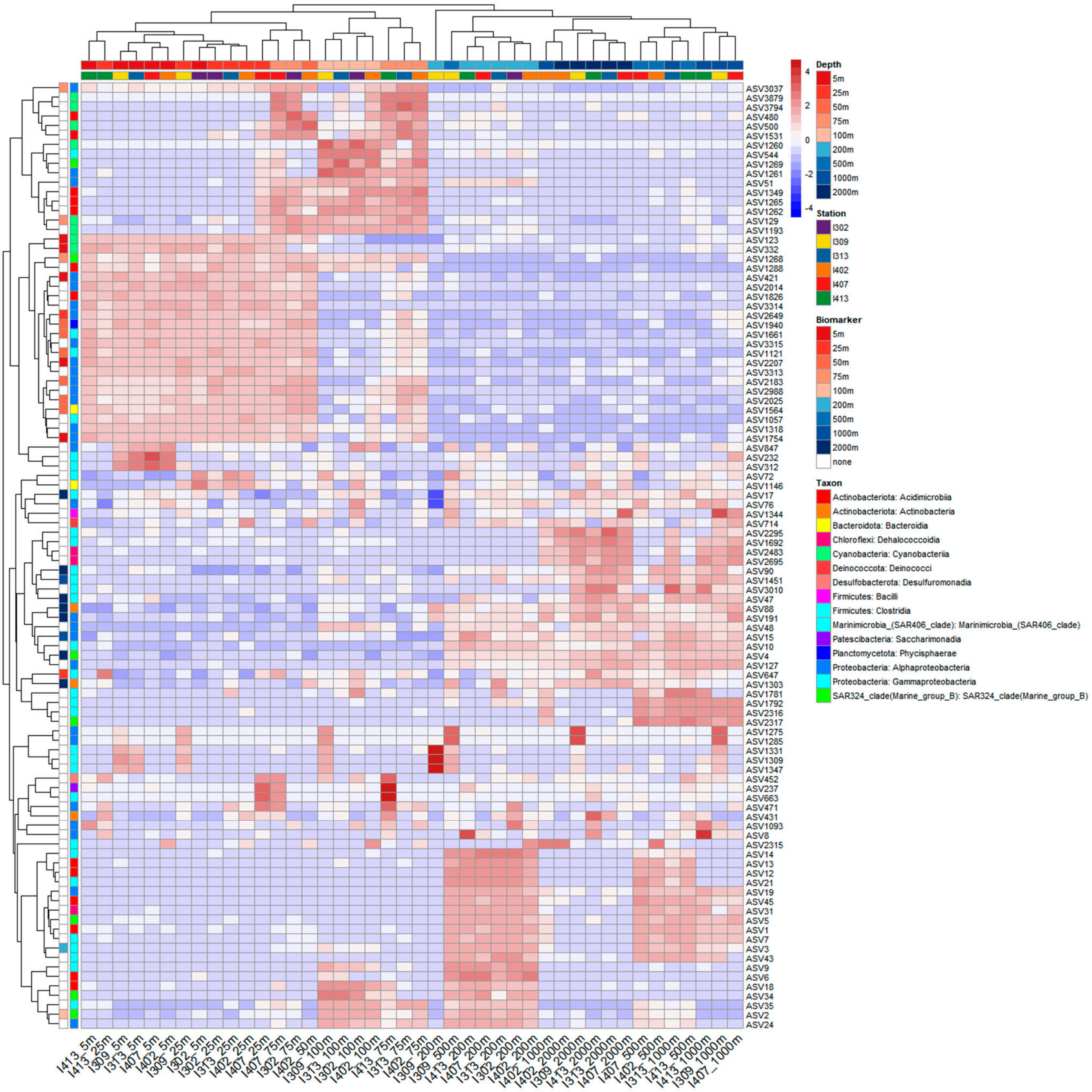

Figure S3. Heatmap of top 100 amplicon sequence variants (ASVs). The relative abundance was log2 transformed and scaled by row. The depths and stations of samples are shown in the top bar. Biomarkers were determined by LEfSe and are shown on the left of the heatmap with the taxonomy at the class level.

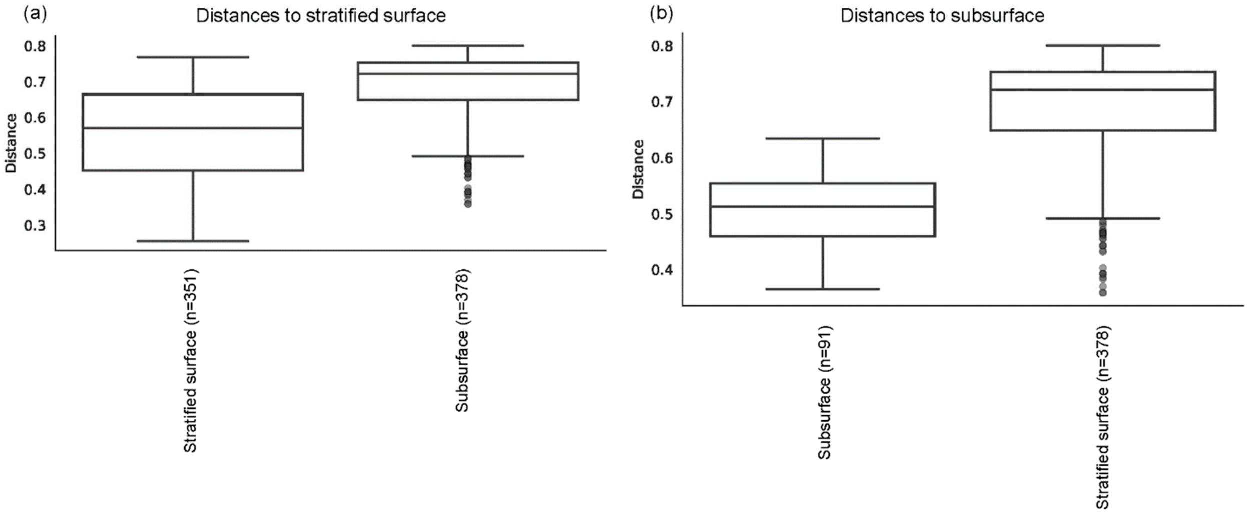

Figure S4. Unweighted UniFrac distance between stratified-surface layer (5 - 200 m) versus subsurface layer (500 -2,000 m). The distance to (a) stratified surface layer and to (b) subsurface layer were presented as the value of Y-axis. The box in the plot represents the interquartile range (IQR), which ranges from the first to third quartiles. The line within the box represents the median. The whiskers extend from the box to the smallest and largest values within 1.5 times the IQR. Any values beyond the whiskers are plotted as individual data points, which may be considered outliers.

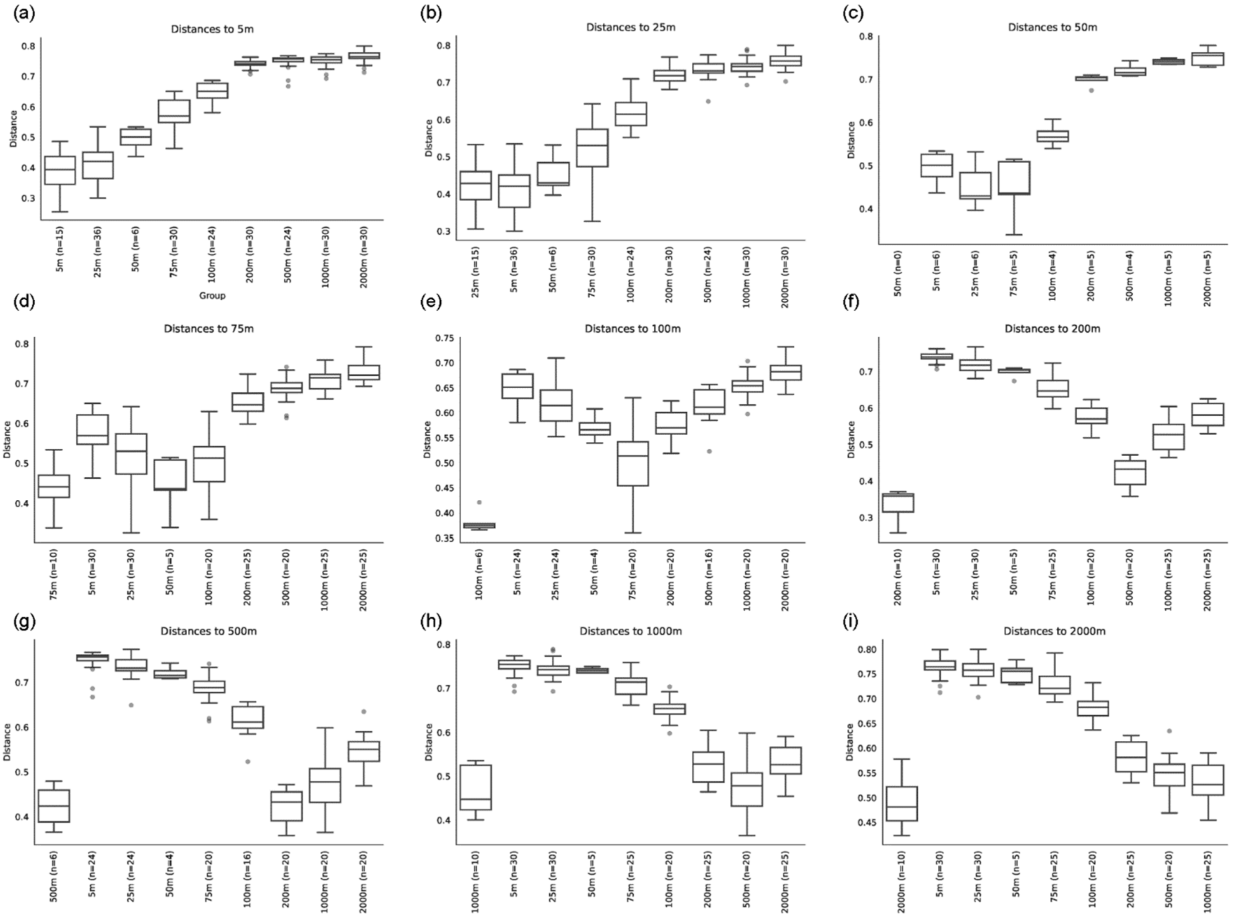

Figure S5. Unweighted UniFrac distance between different depths. The distance of other depths to (a) 5 m, (b) 25 m, (c) 50 m, (d) 75 m, (e) 100 m, (f) 200 m, (g) 500 m, (h) 1000 m, and (i) 2000 m were presented as the value of Y-axis. The box in the plot represents the interquartile range (IQR), which ranges from the first to third quartiles. The line within the box represents the median. The whiskers extend from the box to the smallest and largest values within 1.5 times the IQR. Any values beyond the whiskers are plotted as individual data points, which may be considered outliers.

Table S1. Sampling information about individual stations.

Stations	Sampling Time	Longitude	Latitude
I302	Apr. 16, 2018	80°0.138′E	2°0.029′N
I309	Apr 14, 2018	80°0.059′E	1°59.641′S
I313	Apr 14, 2018	80°0.568′E	3°56.905′S
I402	Apr 15, 2018	79°57.300′E	0°0.535′N
I407	Apr 09, 2018	86°0.023′E	0°0.330′ N
I413	Apr 06, 2018	91°59.654′E	0°0.202′N

Table S2. Information about sampling depths used in environmental and sequencing analyses.

Stations	Sampling depth (m) for environmental analysis	Sampling depth (m) for sequencing analysis
I302	5, 25, 50, 75, 100, 200, 500, 1000, 2,000	5, 25, 75, 100, 200
I309	5, 25, 50, 75, 100, 200, 500, 1000, 2,000	5, 25, 100, 200
I313	5, 25, 50, 75, 100, 200, 500, 1000, 2,000	5, 25, 75, 100, 200, 1000, 2000
I402	5, 25, 50, 75, 100, 200, 500, 1000, 2,000	5, 25, 50, 75, 100, 200, 500, 1000, 2000
I407	5, 25, 50, 75, 100, 200, 500, 1000, 2,000	5, 25, 75, 200, 500, 1000, 2000
I413	5, 25, 50, 75, 100, 200, 500, 1000, 2,000	5, 25, 75, 200, 500, 1000, 2000

Table S3. Information about sampling depths.

Station name	Station	Depth (m)
M15	I302	5
M16	I302	25
M17	I302	75
M18	I302	100
M19	I302	200
M20	I309	5
M21	I309	25
M22	I309	100
M23	I309	200
M24	I309	500
M25	I309	1000
M26	I309	2000
M27	I313	5
M28	I313	25
M29	I313	75
M30	I313	100
M31	I313	200
M32	I313	1000
M33	I313	2000
M34	I413	5
M35	I413	25
M36	I413	75
M37	I413	200
M38	I413	500
M39	I413	1000
M40	I413	2000
M73	I407	5
M74	I407	25
M75	I407	75
M76	I407	200
M77	I407	500
M78	I407	1000
M79	I407	2000
M80	I402	5
M81	I402	25
M82	I402	50
M83	I402	75
M84	I402	100
M85	I402	200
M86	I402	500
M87	I402	1000
M88	I402	2000

Table S4. Relationship (Kruskal-Wallis pairwise test) between the bacterial alpha-diversity indices of different depths.

		5 m	25 m	50 m	75 m	100 m	200 m	500 m	1000 m
	S	0.200185	-						
25 m	E	0.521839							
	R	0.630954							
	S	0.133614	0.133614	-					
50 m	E	0.133614	0.133614						
	R	0.317311	1.000000						
	S	*0.006170	*0.006170	0.769698	-				
75 m	E	*0.006170	*0.006170	0.769698					
	R	*0.044610	*0.035344	0.379775					
	S	*0.010515	*0.033006	0.479500	0.462433	-			
100 m	E	0.055009	0.088082	0.479500	0.327187				
	R	*0.010515	*0.010515	0.157299	0.086411				
	S	0.054664	0.054664	0.317311	0.144127	0.286422	-		
200 m	E	0.054664	0.054664	0.617075	0.273322	1.000000			
	R	*0.016309	*0.016309	0.317311	0.144127	0.393769			
	S	*0.010515	*0.010515	0.157299	0.050044	0.248213	0.831170	-	
500 m	E	*0.010515	*0.010515	0.157299	0.220671	0.563703	0.831170		
	R	*0.010515	*0.010515	0.157299	*0.014306	*0.043308	0.669815		
	S	*0.006170	*0.006170	0.379775	0.174525	0.624206	0.583882	0.327187	-
1000 m	E	*0.006170	*0.006170	0.769698	0.916815	0.624206	0.583882	0.327187	
	R	*0.006170	*0.006170	0.143235	*0.009023	*0.014306	0.361310	0.806496	
	S	*0.010587	*0.044610	0.379775	0.754023	0.327187	0.273322	0.086411	0.347208
2000 m	E	*0.017622	*0.017622	0.769698	0.601508	0.220671	0.201243	0.050044	0.464702
	R	*0.028460	*0.044610	0.379775	0.117185	0.220671	0.201243	0.141645	0.028280

S: Shannon entropy; E: Pielou’s evenness; R: Richness; ‘*’ indicates p-value < 0.05

Table S5. Pairwise PERMANOVA results between stratified layer and subsurface layer.

Group 1	Group 2	Sample size	Permutations	pseudo-F	p-value	q-value
Stratified surface	subsurface	41	999	12.21030219	0.001*	0.001

'*' indicates p-value < 0.05

Table S6. Pairwise PERMANOVA results of different depth.

Group 1	Group 2	Sample size	Permutations	pseudo-F	p-value	q-value
5m	25m	12	999	1.220917182	0.26	0.275294118
5m	50m	7	999	1.986149352	0.144	0.185142857
5m	75m	11	999	5.706940499	0.003*	0.0162
5m	100m	10	999	9.617066323	0.004*	0.0162
5m	200m	11	999	17.1746817	0.002*	0.0162
5m	500m	10	999	12.38177456	0.006*	0.0162
5m	1000m	11	999	12.09474779	0.004*	0.0162
5m	2000m	11	999	11.91216967	0.004*	0.0162
25m	50m	7	999	1.258207341	0.441	0.4536
25m	75m	11	999	3.545340117	0.008*	0.0162
25m	100m	10	999	7.318119481	0.008*	0.0162
25m	200m	11	999	14.34994438	0.005*	0.0162
25m	500m	10	999	10.5995251	0.005*	0.0162
25m	1000m	11	999	10.6944173	0.001*	0.0162
25m	2000m	11	999	10.62257713	0.006*	0.0162
50m	75m	6	999	1.016312344	0.663	0.663
50m	100m	5	999	2.981136642	0.189	0.211636364
50m	200m	6	999	6.329870374	0.151	0.187448276
50m	500m	5	999	3.984407567	0.194	0.211636364
50m	1000m	6	999	3.463190275	0.158	0.1896

Table S6 (continued). Pairwise PERMANOVA results of different depth.

Group 1	Group 2	Sample size	Permutations	pseudo-F	p-value	q-value
50m	2000m	6	999	3.227017643	0.172	0.199741935
75m	100m	9	999	2.935063688	0.016*	0.02304
75m	200m	10	999	9.556167835	0.009*	0.0162
75m	500m	9	999	7.49015692	0.014*	0.021913043
75m	1000m	10	999	7.89087909	0.009*	0.0162
75m	2000m	10	999	7.988232072	0.009*	0.0162
100m	200m	9	999	7.966446457	0.009*	0.0162
100m	500m	8	999	6.311818685	0.03*	0.04
100m	1000m	9	999	6.692054375	0.009*	0.0162
100m	2000m	9	999	6.943694246	0.007*	0.0162
200m	500m	9	999	1.964901956	0.026*	0.036
200m	1000m	10	999	4.342430495	0.007*	0.0162
200m	2000m	10	999	5.473675192	0.007*	0.0162
500m	1000m	9	999	1.603403952	0.016*	0.02304
500m	2000m	9	999	2.799847191	0.012*	0.020571429
1000m	2000m	10	999	2.067319474	0.013*	0.021272727

'*' indicates p-value < 0.05

Table S7. Genera identified as biomarker for individual depth.

Genus	W
Prochlorococcus MIT9313	723
SAR11 clade Ia	723
Candidatus Actinomarina	723
SAR11 clade Ib	722
HOC36	721
Synechococcus CC9902	721
UBA10353 marine group	720
Uncultured Ilumatobacteraceae	720
Uncultured Vicinamibacterales	720
NS4 marine group	719
Chloroplast	718
SAR116 clade	715
BD2-11 terrestrial group	715
Uncultured Microtrichaceae	713
Thioglobaceae (SUP05 cluster)	712
Uncultured Rhodobacteraceae	710
Acidobacteriota (Subgroup26)	707
Dadabacteriales	706
OM75 clade	706
SAR92 clade	705
Uncultured Thiotrichaceae	703
Planctomycetota (Pla3 lineage)	693
NS5 marine group	693
NB1-j	691

Table S7 (Continued). Genera identified as biomarker for individual depth.

Genus	W
Acidobacteriota (Subgroup 21)	690
AT-s3-44	686
AT-s2-59	683
Uncultured Parvibaculaceae	678
SAR86 clade	673
LS-NOB	671
PeM15	671
PS1 clade	670
HgCo23	670
Nitrospina	669
Vicinamibacteraceae	658
NS2b marine group	658
EPR3968-O8a-Bc78	650
Rhodococcus	649
Lentimonas	643
Candidatus Puniceispirillum	642
OM60(NOR5) clade	640
Woeseia	638
Magnetospira	635
Rickettsiales	635
SAR11 Clade IV	633
Uncultured Alphaproteobacteria	631
Coraliomargarita	628
Uncultured SAR11clade I	627
bacteriap25	625
KI89A clade	623
Roseibacillus	619
SAR11 clade III	617

Table S7 (Continued). Genera identified as biomarker for individual depth.

Genus	W
JL-ETNP-F27	613
Nocardioides	599
Uncultured Phycisphaeraceae	598
Pseudoalteromonas	594
Nitrospira	587
Planctomycetes (Pla4 lineage)	583
Lentimicrobiaceae	579

The W value indicate the number of sub-hypotheses that have passed for a given taxa. Taxa with higher W were more significantly abundance in specific depth versus other depths.
